# Cost-effective rapid prototyping and assembly of poly(methyl methacrylate) microfluidic devices

**DOI:** 10.1038/s41598-018-25202-4

**Published:** 2018-05-03

**Authors:** Carlos Matellan, Armando E. del Río Hernández

**Affiliations:** Cellular and Molecular Biomechanics Laboratory, Department of Bioengineering, Imperial College London, London, SW7 2AZ UK

## Abstract

The difficulty in translating conventional microfluidics from laboratory prototypes to commercial products has shifted research efforts towards thermoplastic materials for their higher translational potential and amenability to industrial manufacturing. Here, we present an accessible method to fabricate and assemble polymethyl methacrylate (PMMA) microfluidic devices in a “mask-less” and cost-effective manner that can be applied to manufacture a wide range of designs due to its versatility. Laser micromachining offers high flexibility in channel dimensions and morphology by controlling the laser properties, while our two-step surface treatment based on exposure to acetone vapour and low-temperature annealing enables improvement of the surface quality without deformation of the device. Finally, we demonstrate a capillarity-driven adhesive delivery bonding method that can produce an effective seal between PMMA devices and a variety of substrates, including glass, silicon and LiNbO_3_. We illustrate the potential of this technique with two microfluidic devices, an H-filter and a droplet generator. The technique proposed here offers a low entry barrier for the rapid prototyping of thermoplastic microfluidics, enabling iterative design for laboratories without access to conventional microfabrication equipment.

## Introduction

In the last few decades, microfluidic technologies have arisen as a powerful enabling technology for biomedical research and clinical applications^1,2^. However, the need for cleanroom facilities and advanced microfabrication equipment obstructs innovation in microfluidics. While the advent of soft lithography promoted the widespread of microfluidic technologies, the most common method—PDMS (Polydimethylsiloxane) replica moulding—still requires a photomask and a microfabricated mould, which limits the capacity for iterative design optimization and represents an entry barrier for research groups that want to explore microfluidic tools.

In an effort to make microfluidic research more accessible and cost-effective, thermoplastic materials such as polymethyl methacrylate (PMMA)^3^, cyclic olefin copolymer (COC)^4^ or polycarbonate (PC)^5^ have gained interest as an alternative to conventional materials, particularly for commercially-oriented applications. These materials present better mechanical properties than PDMS and are more robust and easier to manufacture than traditional materials such as silicon (Si) or glass. They can be fabricated using a variety of techniques^6^; including microinjection moulding^7^, hot embossing^8^, casting, reactive ion etching, and mechanical (milling)^9^ or laser micromachining^10^; making them easier to standardise and handle, and amenable to industrial manufacturing.

Among the different fabrication methods, CO_2_ laser micromachining offers high versatility and cost-effective manufacturing^10^. Commercially available benchtop laser cutters can be used to generate a large variety of microfluidic designs with different specifications without the need for moulds or specialized equipment, thus enabling rapid prototyping and iterative design of microfluidic devices. Tuning of the laser characteristics (power, speed and focal distance) allows control of feature depth, width and geometry, highlighting the versatility of the technique. The main downside of laser ablation processes is the low optical quality of the micromachined surface. The melting, vaporization and ejection of the material caused by the incident laser irradiation promotes the formation of pores and the deposition of material residue, generating high surface roughness and limiting the applicability of laser-cut devices.

Conventional treatments to reduce surface roughness (annealing) require lengthy and complex thermal cycles above the glass transition temperature of the thermoplastic^11^. These high temperatures can cause significant warping of the bulk material, deformation of the channel geometry and loss of feature fidelity. As an alternative to thermal annealing, solvent-based methods have been studied. These methods employ solvents such as chloroform^12^, acetone^13^ or cyclohexane^12^ to directly reduce the roughness of the ablated surface and to acts as plasticisers, reducing the glass transition temperature (Tg) of the surface to enable additional thermal remodelling of the polymer chains. Solvent treatment followed by thermal cycling, however, can cause crazing of the surface of the thermoplastic through solvent-stress cracking and thermal propagation. Optimization of the treatment protocol is therefore critical to achieve a suitable surface quality for optical clarity without cracks.

Effective and stable bonding of thermoplastics is another barrier for rapid assembly of microfluidic devices and high throughput commercial manufacturing. Thermal bonding^14^, solvent bonding^15^, adhesive bonding, plasma and other surface activation treatments^16^ and microwave bonding^17^ have all been explored. Thermal bonding is the most common method for thermoplastic materials like PMMA due to its accessibility and simplicity, but the high temperatures (above 105 °C) can distort the device geometry.

Solvent-assisted thermal bonding exploits the use of plasticisers, which reduce the glass transition temperature of the thermoplastic surface, to achieve thermal bonding at lower temperatures and shorter times. Ethanol^18^, chloroform^19^, dibutyl phthalate (DBP)^20^, and DMSO/Methanol^21^ have been reported as effective plasticisers for rapid solvent-assisted thermoplastic bonding. While these methods allow for efficient assembly of thermoplastic devices without causing bulk distortion, they present some limitations. Some of the solvents employed are hazardous and highly cytotoxic, and large quantities can cause residual deposits of dissolved material on the channels^18^.

Furthermore, these methods are only effective for thermoplastic-thermoplastic bonding, but a number of microfluidic applications require bonding to specific substrates for their chemical, optical or electrical properties, including glass, silicon, or piezoelectric materials such as LiNbO_3_. Indirect or adhesive bonding can offer higher material compatibility, but difficulty in achieving uniform and controlled adhesive delivery is a significant limitation of these methods, which include adhesive printing^22^, capillary filling^23^ and gas injection^24^.

Here we develop an integrated and highly versatile manufacturing process for PMMA microfluidics, including laser machining, surface treatment and bonding. We explore the effect of an unfocused laser configuration under different power and focal distance combinations on the channel geometry. A solvent vapour treatment method based on readily available and conventional solvents was optimized to reduce the surface roughness induced by the laser ablation process. The use of low temperatures (70 °C, below the normal Tg of PMMA) ensures minimal deformation of the bulk material. This technique enhances optical clarity, enabling visualization of particles and culture of cells inside the channels. We also present a capillarity-assisted, room-temperature, low pressure, adhesive-based bonding technique. Bonding can be achieved at room temperature and no bonding pressure is required, preserving the device architecture throughout the process. The bond is stable over long time and can withstand high hydraulic pressures and flow rates above those commonly used in microfluidics. This method is successful in bonding PMMA devices to glass, silicon, LiNbO_3_ and PMMA. We illustrate this method with two microfluidic devices: an H-filter and a T-junction droplet generator. The techniques presented here enable inexpensive translation from idea to prototype and low-cost, low-requirement fabrication of devices to facilitate the adoption of microfluidic tools by new research groups.

## Materials and Methods

The rapid and cost-effective fabrication method for PMMA microfluidics developed here consists of three main processes: laser micromachining of the PMMA devices, surface treatment to improve surface quality and capillarity-assisted adhesive bonding for assembly.

### Laser micromachining

A benchtop CO_2_ (10.6 μm) laser cutter (VLS2.30, Denford & Universal Laser System, USA) with a maximum power of 30 W and a z-adjustable stage was used to laser-machine the surface of 3 mm thick cast polymethyl methacrylate (PMMA) sheets (CRYLUX Polycasa and Perspex, supplied by the Imperial College Advanced Hackspace). The protective film was removed on the engraved side of the PMMA sheets prior to the laser-machining to avoid interfering with the laser ablation process.

Microfluidic channels were designed in CorelDRAW Graphic Suite X7 and converted to laser paths with the laser cutter built-in software. Laser parameters were controlled with this software by adjusting the laser power and the z-position of the cutting stage. Channels were engraved under different combinations of laser power (10, 20, 30, 40, 50, 60, 70, 80% of the maximum power of 30 W) and distance-to-focus (DF) measured as the distance between the stage and the focal point of the laser (3, 5, 10, 15, 20 and 40 mm, with a minimum of 3 mm corresponding to a focused laser configuration).

Prior to treatment or analysis, engraved PMMA chips were washed with isopropanol (IPA) to eliminate polymer residue, sonicated in DI water for 2 minutes, rinsed in IPA and dried with clean N_2_ gas. Channel geometry was characterised by contact profilometry (2.5 μm tip radius, 3.33 μm resolution, 5 mg force, 540 μm measurement range) with a stylus profilometer (Dektak 150, Veeco, USA) and channel dimension data was extracted with MATLAB (Mathworks, USA). The effect of distance-to-focus on the channel cross-section morphology was analysed by scanning electron microscopy (SEM). SEM samples were engraved (30% power, 10% speed) at different DF (5, 10, 15 and 20 mm), and treated with acetone vapour for 5 minutes followed by thermal treatment for 10 minutes. Samples were cleaned as described here and gold-coated *via* sputtering for 30 seconds at 20 mA (K575X Sputter Coater, Quorum Technologies, UK) prior to being analysed in a scanning electron microscope at 20 kV operating voltage (JEOL JSM-5610LV, Jeol, UK).

### Solvent vapour surface treatment

To uniformly expose the PMMA channels to acetone, a custom-built vapour chamber with a metal platform and an acetone reservoir was constructed. PMMA chips were cleaned as described, taped (double-sided Kapton tape, Agar Scientific) to glass slides and placed on the metal platform with the engraved channels facing the reservoir of acetone. The chamber was closed with a glass lid and the acetone was allowed to evaporate.

The engraved PMMA chips were exposed to acetone vapour in this manner for 3, 5 and 10 minutes at room temperature (~18 °C), 25 °C or 30 °C. The temperature was controlled throughout the process in an incubator, and the acetone reservoir was pre-heated for 5 minutes before exposing the engraved devices. Immediately after exposure to the acetone vapour for the specified time, PMMA chips were heat-treated at 70 °C for 20 minutes in an oven and then allowed to cool down to room temperature.

Surface topography of engraved channels treated with this combination of acetone vapour and thermal annealing was assessed by scanning electron microscopy (SEM). Channel widths of devices treated under different temperature and exposure time conditions were extracted from scanning electron micrographs, with at least 10 measurements per sample, and compared via one-way ANOVA with Tukey post-hoc analysis.

Contact angles for bare PMMA (before laser ablation), untreated PMMA and surface treated PMMA (after exposure to acetone at 25 °C for 5 minutes and thermal treatment at 70 °C for 20 minutes) were measured with a Drop Shape Analyser DSA100 (Krüss, Germany). Static contact angles were measured with 2 µl drops using the sessile-drop method with a minimum of 6 measurements per sample and three repeats.

### Adhesive bonding

A two-part epoxy resin (Araldite Standard) was mixed according to the manufacturer (1:1) and diluted with acetone (~1 g/ml) by stirring until the adhesive mix was homogeneous. Clean PMMA chips were positioned and aligned with transparent tape on the substrate prior to bonding. No additional pressure was applied to the assembly while bonding. PMMA (CRYLUX, Polycasa, UK), glass (Thermoscientific, UK), silicon and LiNbO_3_ (University Wafer, USA) were used as substrates. The adhesive mix was injected with a needle (BD Microlance 3, 21 G × 1/2″, BD, UK) in a controlled manner at the edge between the PMMA device and the overhanging substrate, and it immediately flowed into the interstitial space between the bonding surfaces due to capillarity forces. To avoid overflowing the channels, the adhesive front was visually inspected and used as reference to control the amount of adhesive delivered. The adhesive was allowed to cure overnight at room temperature before testing for channel leaking.

To analyse the bonding at the interface between the PMMA chip and the substrate, standard samples were fabricated by two passes of laser ablation (40% power, 15 mm distance-to-focus, 650 μm separation between adjacent paths) and treated with acetone vapour as previously described for 5 minutes at room temperature followed by a heat treatment at 70 °C for 15 minutes. The treated PMMA chips were bonded to glass and PMMA substrates respectively according to the method presented here and allowed to cure for 48 hours before laser sectioning.

Transverse sections of the assembled devices were laser-cut (100% power, 10% speed, 4 mm distance-to-focus (DF) for the glass-bonded samples and 100% power, 1.5% speed, 6 mm DF for PMMA-bonded devices) to a thickness of 5 mm to analyse the cross-section of the device. These transverse sections were gold-sputtered (30 s, 20 mA) and analysed by scanning electron microscopy at a working voltage of 20 kV (JEOL 5610LV).

Bonding strength was analysed according to the standard for shear strength of adhesively-bonded rigid plastics (ASTM D3163). Standard PMMA (Perspex) samples (101.6 mm × 25.4 mm, length x width) with a thickness of 3 mm were adhesively bonded to each other with a total overlap area of 25.4 mm × 25.4 mm and allowed to cure for 72 hr or 2 months prior to testing. For comparison, PMMA-PMMA specimens were treated with ethanol, clamped and thermally bonded at 90 °C for 30 min. The lap-shear joint tests were carried out in an Instron 3366 Dual Column universal testing machine (Instron, UK) with a 10 kN load cell at a cross-head speed of 1.27 mm/min.

### Example microfluidic devices

An H-filter and T-junction droplet generator were fabricated by laser engraving on PMMA at 30% power, 15 mm DF and 10% speed as described in section 2.1. The main channel of the droplet generator was engraved by two parallel passes of the laser beam with a separation of 625 µm, resulting in a single, wider channel. Devices were treated with acetone vapour for 5 minutes at 25 °C, followed by heat treatment at 70 °C for 20 minutes prior to bonding to reduce surface roughness as described in section 2.2. Devices were bonded to glass slides using the capillarity-assisted adhesive bonding method described in section 2.3.

A dual syringe pump (PHD2000 Infusion, Harvard Apparatus, USA) was used to operate the devices, which were connected to the syringes with microfluidic tubing (Tygon Lab Tubing, Non-DEHP, 1/32′′ID × 3/32′′OD, Cole-Parmer, USA). Access ports (inlets/outlets) were laser cut at 100% power, 3% speed, 3 mm DF. With these conditions, a diameter of 2.1 mm was determined to provide the best fitting for the tubing used in these experiments. In H-filter devices, microfluidic tubing was directly inserted in the inlet and outlet ports, producing a pressure seal between the tubing and the PMMA device without collapsing the tube. In T-junction devices, larger (3 mm) access ports were laser cut into the device, and PMMA connecting rings with internal and external diameters of 2.1 mm and 4 mm respectively were glued with an epoxy resin (Araldite Crystal) over the ports. Microfluidic tubing was inserted through these rings and similarly glued to them to produce a permanent seal without clogging the tubing.

The H filter was operated with two 10 ml plastic syringes (Terumo, SS-10S), connected directly to the inlets of the device. A sample containing a 0.1% (w/v) solution of Brilliant Blue R (Sigma-Aldritch, UK) in deionised water and a 0.1% (v/v) solution of 20 µm polystyrene beads (density 1.05 g/cm^3^, Sigma-aldritch) was injected through the “sample” inlet at flow rates of 5, 10 and 15 ml hr^−1^, and deionised water was simultaneously injected in the opposite inlet as a buffer at the same flow rate. Fluid from the two outlets was collected and analysed for particle contents and dye concentration. Particle contents of the two outlets were analysed with a haemocytometer, while dye concentration was analysed via colourimetric assay in a plate reader (wavelength 595 µm, 5 second measurement, 35 µl sample volume. Infinite F50, Tecan, UK). Images of the H filter operation were captured at 1 ml hr^−1^ flow rates with a microscope camera (Moticam MP 5.0, Motic, Germany).

The T-junction droplet generator was operated with a 10 ml syringe for the dispersed phase and a 20 ml syringe for the continuous phase (Terumo, SS-10S), resulting in a flow rate ratio between the continuous and dispersed phases (${Q}_{C}/{Q}_{D}$) of 1.63 due to the difference in syringe diameter (15.8 mm and 20.15 mm for the dispersed and continuous phases respectively). The device was operated as a water-in-oil droplet generator, with deionised water injected in the side channel and vegetable oil injected in the main channel. Images of the device operation were captured at 0.2 ml hr^−1^, 0.5 ml hr^−1^and 1 ml hr^−1^ flow rates (dispersed-phase flow rate, Q_D_) with a microscope camera (Moticam MP 5.0, Motic, Germany), and the videos were analysed (ImageJ) to determine the average droplet size and polydispersity index (calculated here as the percentage of the standard deviation of the particle diameter normalised by their mean diameter).

### Statistical Methods

All statistical analyses were conducted with the Prism graphical software (GraphPad Software). Data were generated from a minimum of 3 repeats and analysed to obtain the mean values and standard error of the mean (s.e.m) or standard deviation (SD) as indicated in the text. Statistical comparisons for significance were conducted *via* unpaired t-test (for two groups), one-way ANOVA with post-hoc Tukey test (for multiple groups) or two-way mixed ANOVA with Bonferroni post-test (for multiple groups with two factors) as indicated in the text. Statistical significance was defined as *p < 0.05, with additional symbols **p < 0.01 and ***p < 0.001.

### Data Statement

All data in this work are available directly from authors.

## Results

### Characterisation of laser micromachining

Laser micromachining is a subtractive technique that uses a computer-controlled CO_2_ laser to ablate the surface of a thermoplastic. Microfluidic channels are engraved on the surface according to computer-designed laser paths, eliminating the need for a mask or mould during the fabrication process. Moreover, control of the channel dimensions can be achieved by tuning two laser parameters: laser power and distance-to-focus (DF), i.e. the distance between the focal point of the laser and the substrate. As a result, this manufacturing method offers a very high versatility, and enables iterative rapid prototyping and sharing of microfluidics designs.

**Figure 1:**
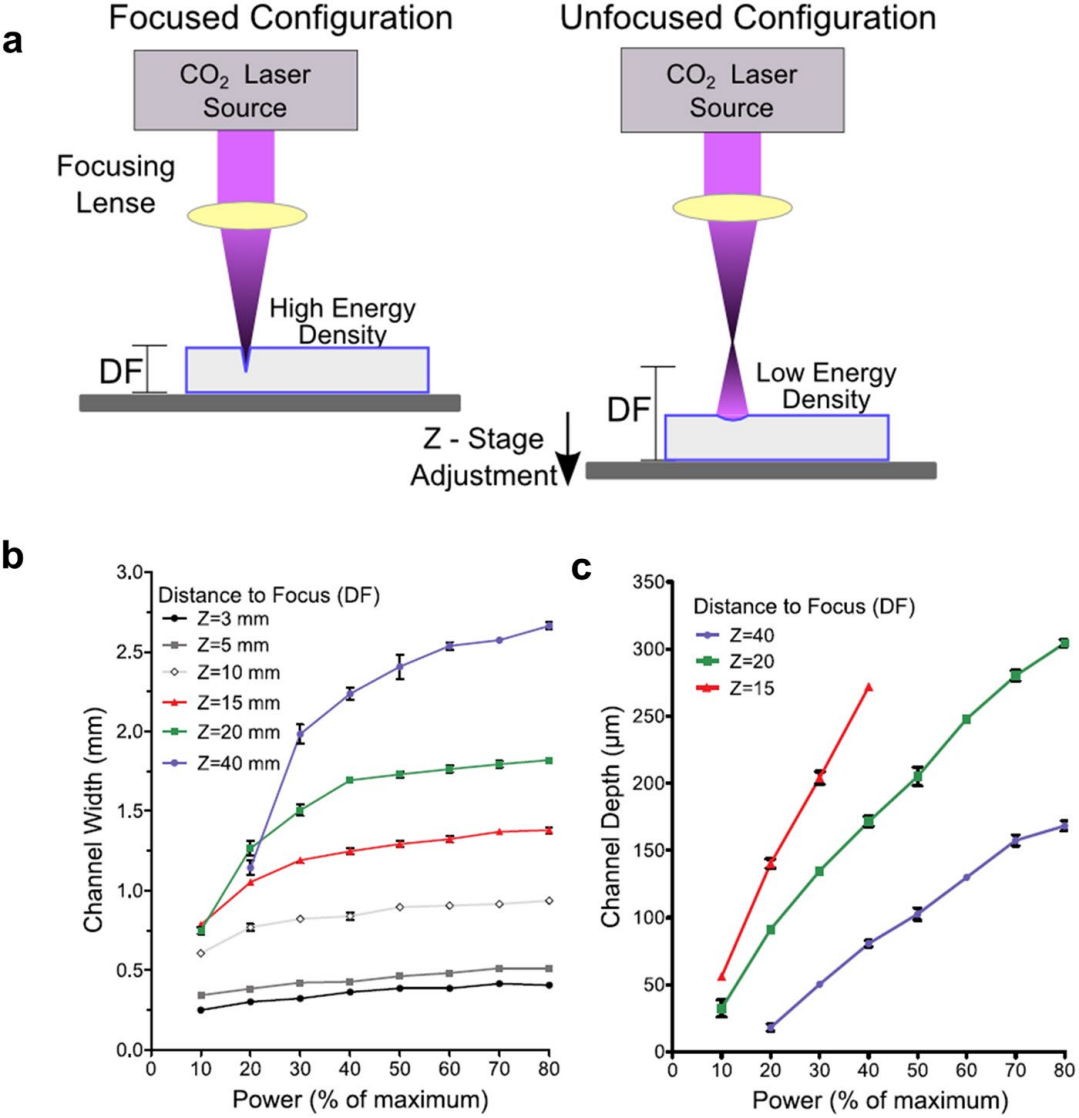
Characterisation of the laser ablation. (**a**) Laser ablation process in the focused and unfocused configuration. Increasing the distance between the focal point and the surface of the material causes the laser energy to be spread over a wider area, resulting in lower energy density. Adjustments of the stage and laser source enable control of the laser. Channel width (**b**) and depth (**c**) of channels engraved in PMMA resulting from different combinations of laser power (as percentage of the maximum 30 W) and distance-to-focus (3, 5, 10, 15, 20 and 40 mm) as extracted from profilometric analysis (mean ± SD, n = 3).

Profilometric analysis of the engraved channels revealed a wide range of dimensions resulting from different laser configurations (Fig. 1), with channel widths ranging from ~250 μm to ~2.5 mm, and channel depths ranging from ~20 μm to over 300 μm (at high power/high focus configurations, the high aspect ratio of the channels prevented accurate profilometric analysis). Laser power was found to correlate positively with channel depth, following a near linear relationship for all distances-to-focus explored. At all distances-to-focus, channel width was also observed to increase in correlation with laser power, although this trend was found to depend on the DF, with a much larger increase at high distances to focus. In relative terms, the change from 20% to 30% power, for instance, resulted a ~7% increase in width in the focused laser configuration and a ~73% increase in the 40 mm DF configuration.

**Figure 2:**
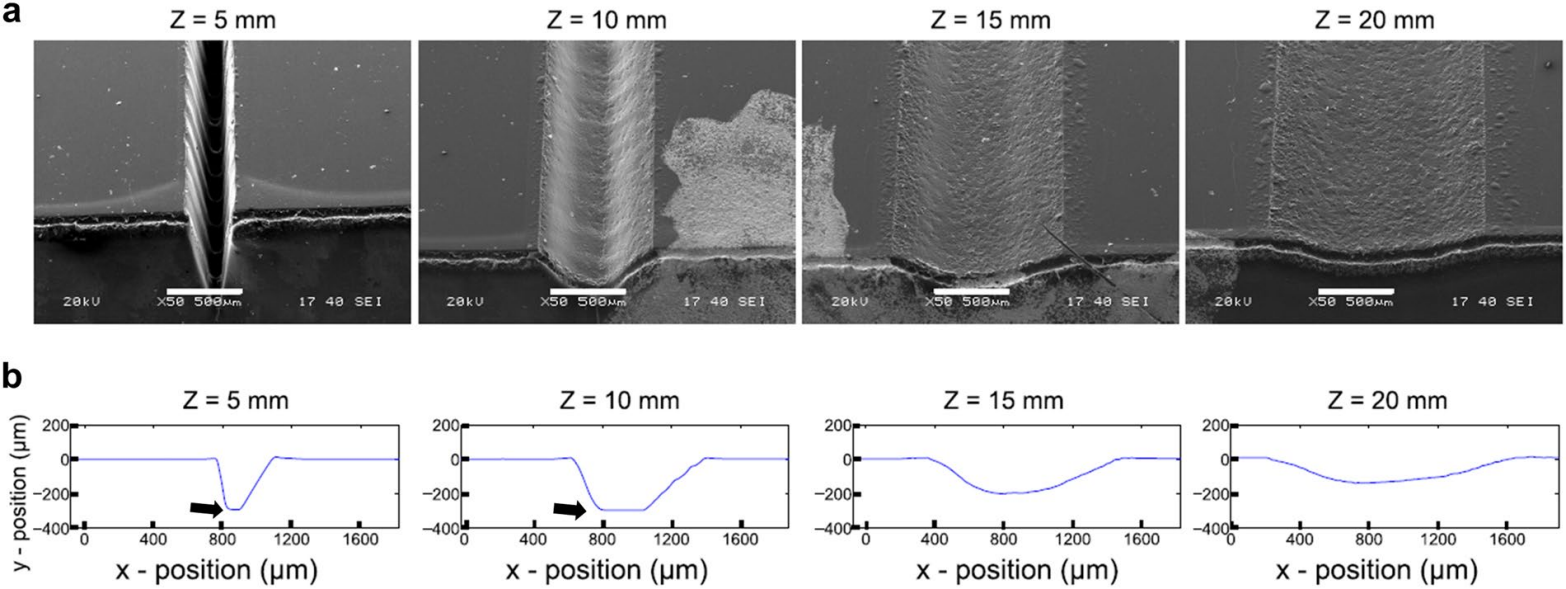
Effect of distance-to-focus on channel geometry. Cross-section and morphology of microfluidic channels laser-engraved (30% power, 10% speed) at varying distance-to-focus (Z = 5, 10, 15 and 20 mm respectively). (**a**) Scanning electron microscopy of the engraved channels (tilt 45°) reveals high aspect ratio (deep and narrow) channels at low distances, and low aspect ratio (shallow and wide) channels at higher distances. Scale bar: 500 μm. (**b**) Profilometer analysis of the microfluidic channels reveals changes in cross-section (from a narrow gaussian morphology to semicircular) with increasing level of laser unfocusing. Axes show real aspect ratio. For the 5 mm and 10 mm FD channels, the stylus profilometer reaches the limit of its measurement range, resulting in a flat profile curve (black arrows).

For any given laser power, a higher degree of unfocusing (i.e. higher distance to focus) resulted in wider and shallower (lower depth) channels. For the 40 mm DF, the lowest laser power (10%) resulted in no measurable channel ablation on PMMA. Consistent with the profilometric data, electron microscopy analysis of the microfluidic channels revealed channel morphology and aspect ratio to be dominated by the degree of unfocusing (Fig. 2). Focused laser configurations (low DF) produced channels with a deep and narrow cross-section (high aspect ratio), whereas highly unfocused configurations resulted in shallow, semi-circular channels of low aspect ratio, offering high flexibility.

### Surface Treatment

**Figure 3:**
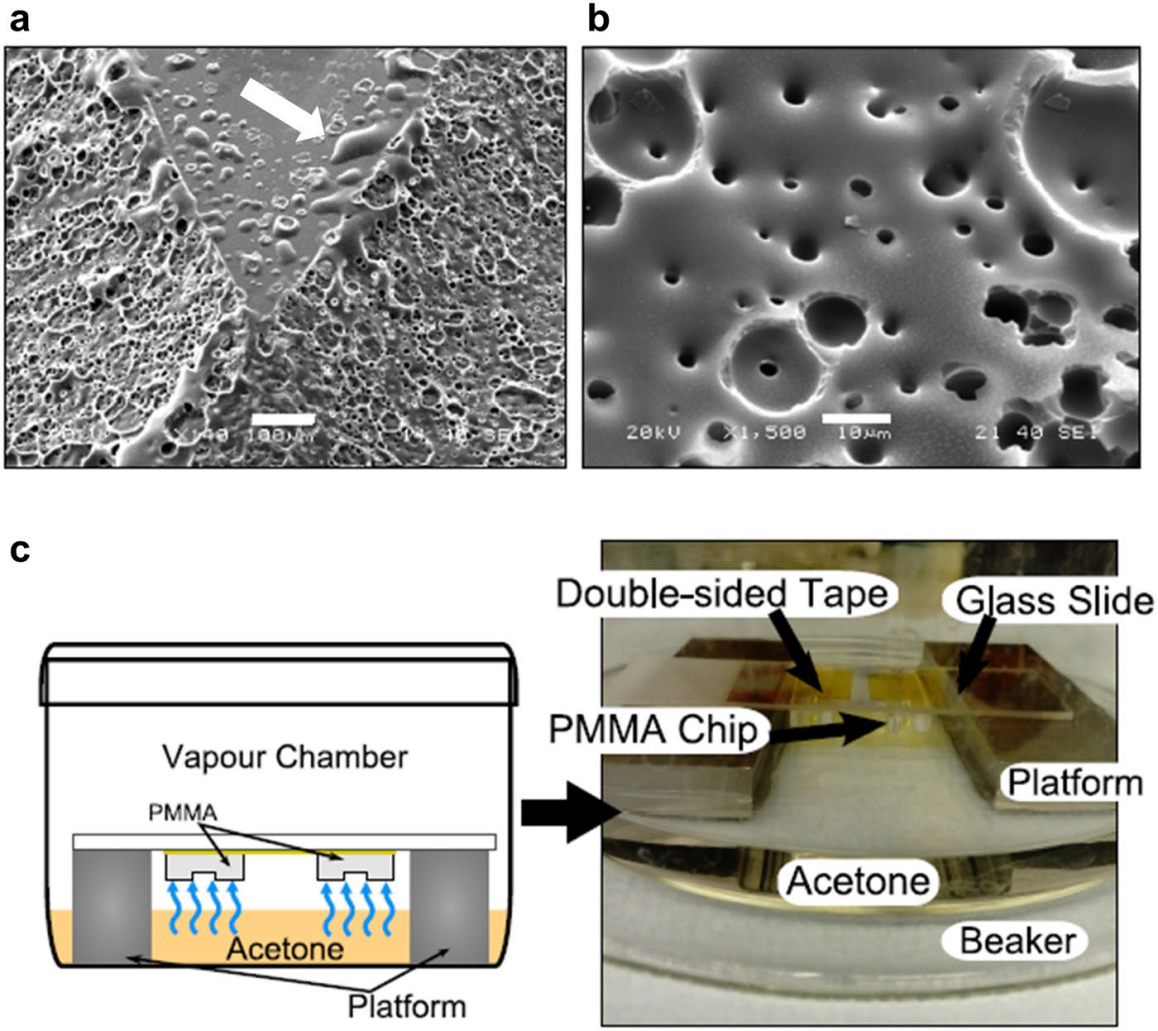
Surface Roughness and treatment setup. (**a**) Surface defects appearing on an engraved flow splitter. Material residue from the ablation process deposits on flat surfaces (white arrow) and can cause leaking. Scale bar: 100 μm. (**b**) Detail of the microcavities and pores formed during ablation of the surface of the channel. Scale bar: 10 μm. (**c**) Acetone vapour treatment chamber setup. The engraved PPMA channels are exposed to the acetone vapour as it evaporates from a reservoir, while a metal platform prevents direct contact with the liquid solvent.

While offering a high potential for rapid prototyping of microfluidic devices, the laser ablation process generates a high surface roughness through the formation of microcavities and other surface irregularities (Fig. 3a,b). The poor optical clarity of these surfaces, together with the increased adsorption and biofouling, limits the applicability of laser-engraved thermoplastic microfluidic devices. In order to improve the optical and surface quality, post-engraving processing of the surface was found to be a necessary step prior to application of the microfluidic device. In order to remodel the engraved surface, channels were exposed to acetone vapour under different conditions of temperature and exposure time in a custom-made vapour chamber (Fig. 3c) followed by thermal treatment.

**Figure 4:**
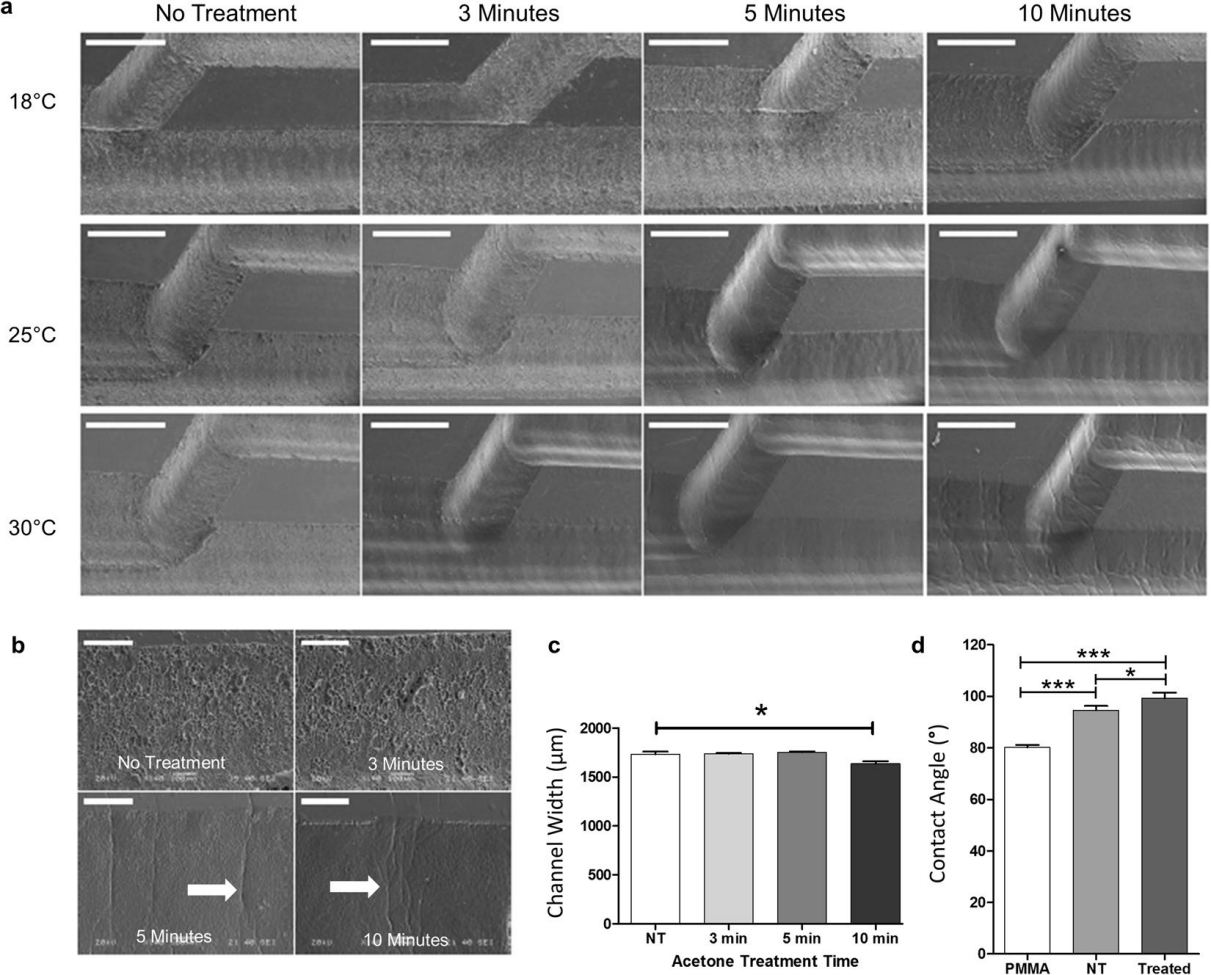
Surface treatment of engraved channels. (**a**) Scanning electron microscopy of channel surface for PMMA laser-machined devices treated with acetone vapor for 3, 5 and 10 minutes at 18 °C, 25 °C or 30 °C, followed by thermal treatment at 70 °C for 20 minutes, as compared to samples with no treatment. Scanning electron micrographs reveal changes in the surface roughness and presence of microcavities and other defects. Scale bar: 1000 μm. (**b**) Detail of channel walls after acetone vapour exposure for 3, 5 and 10 minutes at 25 °C. Cracks can be observed on the surface (white arrow). Scale bar: 200 μm. (**c**) Changes in channel width after exposure to acetone vapour at 25 °C for different times followed by thermal treatment (20 minutes, 70 °C for all samples). Channel width was analysed from scanning electron micrographs. Data analysed by one-way ANOVA (mean ± s.e.m, n = 3, *p < 0.05). (**d**) Contact angle measurements for bare PMMA, and laser-cut PMMA without treatment (NT) and after acetone-assisted thermal treatment. (*p < 0.05, ***p < 0.001). Data analysed by one-way ANOVA (mean ± SD, n = 3) with Tukey post-hoc test.

For the lowest acetone vapour treatment temperature explored (18 °C), only a minimal improvement of the surface roughness was observed after an exposure time of 10 minutes, while no effect was observed for shorter treatment times (Fig. 4). At a exposure temperature of 25 °C, smooth channels were observed after 5 and 10 minutes of exposure to the acetone vapour followed by thermal annealing, whereas the highest temperature (30 °C) resulted in remodelling of the surface even with a short (3 minutes) solvent exposure. For the longer treatments, formation of cracks and crevasses was observed on the surface of the channels, particularly at 30 °C and for exposure time of 10 minutes.

Significant changes in channel dimensions after treatment were only observed for 10 minutes exposure at 25 °C, (Fig. 4c), with a reduction in channel width of ~90 μm as compared to untreated samples (initial channel width 1730 ± 30 μm), which represents a ~5% change in dimension. For the remaining treatment configurations, the channel dimensions were conserved with no significant changes (Supplementary Fig. S1).

The water contact angle of PMMA (80.2° ± 0.6°, Mean ± s.e.m, n = 3) was found to increase after laser ablation (Fig. 4d). Moreover, an increase in the contact angle was observed after treating the laser-ablated surface with solvent vapour followed by thermal remodelling, from 94.6° ± 1.0° to 99.3° ± 1.3° (Mean ± s.e.m, n = 3).

### Adhesive bonding

A cost-effective, accessible, high throughput assembly method for PMMA microfluidic devices is a fundamental requirement for the widespread use of these technologies. Adhesive bonding methods offer higher flexibility with the substrate material, are less disruptive to the device architecture than conventional thermal fusion, and can be carried out with minimal equipment. However, delivering the adhesive homogeneously at the interface without causing channel clogging remains a central challenge in adhesive-based bonding.

**Figure 5:**
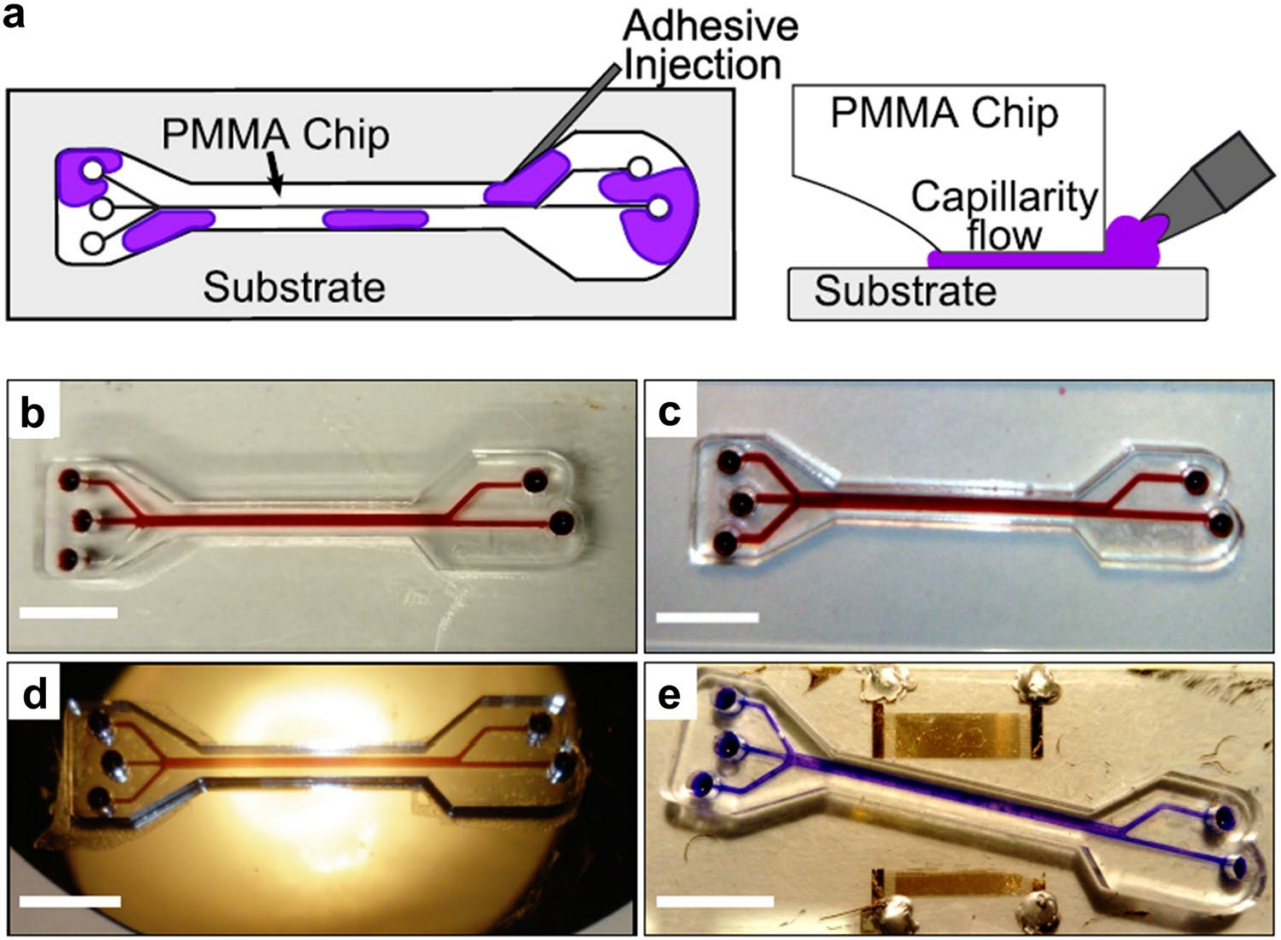
Capillarity-assisted adhesive bonding. (**a**) Schematic of the adhesive delivery method. The adhesive mix is injected into the interstitial space between the chip and the substrate, and capillarity forces drive the flow of the adhesive throughout the bonding surfaces without flooding the channels. (**b**–**e**) PMMA microfluidic devices adhesively-bonded via capillarity-assisted adhesive delivery on PMMA (**b**) glass (**c**) silicon (**d**) and LiNbO_3_ (**e**) substrates. Scale bar: 1 cm.

The capillarity-assisted adhesive bonding method explored resulted in an effective seal between the bonding surfaces of the PMMA chip and substrate, and was found to be more controllable and less prone to channel clogging and blockage than other adhesive application methods explored (Supplementary Fig. S3) including adhesive stamping and injection through access holes. This technique was effective in bonding laser-engraved PMMA devices to PMMA, glass, silicon and LiNbO_3_ substrates (Fig. 5), with no leaking or delamination observed with a flow rate of 250 μl min^−1^.

**Figure 6:**
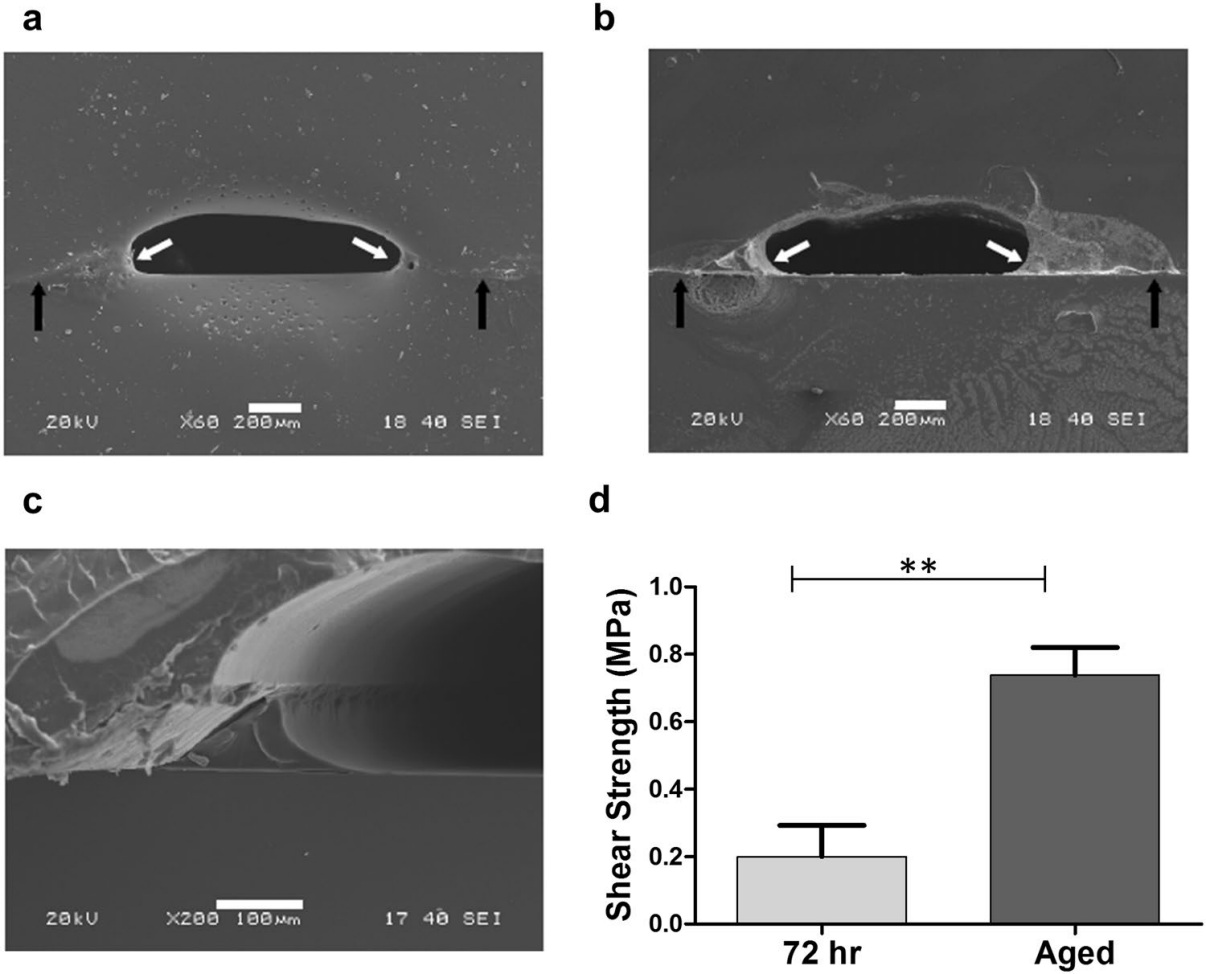
Characterisation of the adhesive bond. Transverse section PMMA chips bonded to PMMA (**a**) and glass (**b**) substrates analysed by scanning electron microscopy. A thin layer of adhesive can be observed at the interface between the two materials (black arrows). An accumulation of adhesive at the edges of the channel, resulting in an oval cross-section, can also be observed (white arrows). Scale bar: 200 μm. (**c**) Detail of the accumulation of adhesive at the edges of the channel in a PMMA-Glass substrate. Scale bar: 100 μm. (**d**) Bond shear strength for PMMA-to-PMMA adhesive bonding via capillarity-assisted adhesive delivery. Samples were allowed to cure for 72 hours or 2 months (aged) before tensile testing. **p < 0.01 (Mean ± SD, n = 3).

Scanning electron microscopy analysis of the cross-section of PMMA channels bonded to PMMA (Fig. 6a) and glass (Fig. 6b) revealed an intermediate layer between the two materials, corresponding to the adhesive. Some degree of fusion between PMMA layers was also found in the PMMA-PMMA bonded devices. With both substrate materials, adhesive was observed to accumulate on the edges of the channels (Fig. 6c) forming a concave shape that resulted in an oval cross-section channel as opposed to the initial gaussian morphology of the engraved channels.

The bond strength of the capillarity-assisted adhesive bonding was characterised by a standard adhesion shear strength test (ASTM D3163). PMMA-PMMA samples bonded by capillarity-assisted adhesive bonding showed a shear strength of 200 ± 92 kPa when cured for 72 hours, and 738 ± 82 kPa (mean ± SD, n = 3) when allowed to cure for 2 months (Fig. 6d). In the specimens cured for 72 hours, partial debonding through cohesive failure was observed, with residual material remaining adhesive between the two bonding surfaces, while samples cured for 2 months showed clean debonding with no remaining adhesiveness, resulting in significantly higher bonding strength. In comparison, lap joint shear testing of thermally bonded PMMA-PMMA samples resulted in failure of the specimen at a load of 1084 ± 86 N (mean ± SD, n = 3), significantly higher than the load required for cohesive failure of the adhesive bond (Supplementary Fig. S4).

### Microfluidic example devices

Two simple microfluidic devices were fabricated as a proof-of-concept of the rapid prototyping method developed: and H-filter and a T-junction droplet generator.

**Figure 7:**
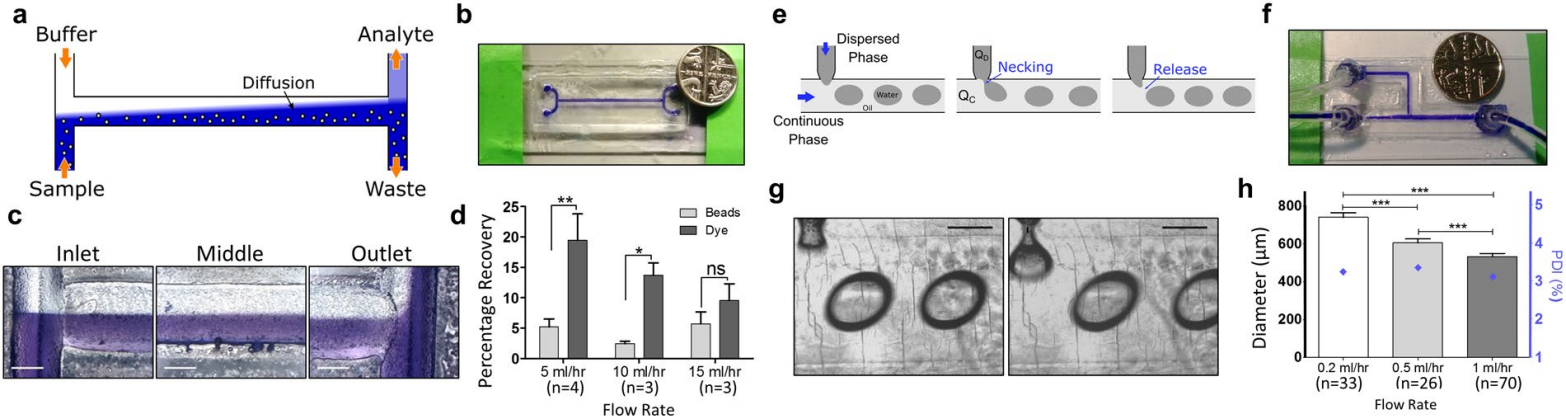
Example microfluidic devices. (**a**) Schematic of the operation of an H-filter. Diffusion dominates mass exchange between parallel streams, enabling size fractioning via flow splitting. (**b**) PMMA microfluidic H-filter fabricated by laser ablation and capillarity-assisted adhesive bonding. Coin (5 pence) for comparison. (**c**) Operation of the PMMA H-filter under laminar flow. The bottom stream (sample inlet) contains a blue dye and 20 µm polystyrene beads. The sample and buffer streams remain parallel and split at the outlet, with a fraction of the dye that has diffused to the buffer stream exiting through the analyte outlet. Scale bar: 500 μm. (**d**) Percentage of the dye and beads contents recovered at the analyte outlet of the H filter at different flow rates (5, 10 and 15 ml hr^−1^ ). Data analysed by two-way mixed ANOVA (*p < 0.05, **p < 0.01 and ns non-significant) with Bonferroni post-test and shown as mean ± s.e.m. (**e**) Schematic of the droplet generation process in the T-junction. The interaction between the two immiscible phases causes necking and pinching of the dispersed phase and release into the main channel. (**f**) PMMA T-junction droplet generator with glued tubing. Coin (5 pence) for comparison. (**g**) Formation and necking of a water-in-oil droplet at the T junction via dripping regime. Scale bar: 500 µm. (**h**) Mean droplet diameter and polydispersity index (PDI) for the droplets produced in the T-junction droplet generator at different flow rates: 0.2, 0.5 and 1 ml hr^−1^ (flow rate ratio between continuous and disperse phases Q_C_/Q_D_  = 1.63 in all cases). Droplet diameter data analysed by one-way ANOVA (***p < 0.001) with Tukey post-hoc test, and shown as mean ± SD.

The H-filter consists of two inlets that join into a central channel and then split into two outlets. This device exploits the laminar properties of microfluidic flow to prevent convective mixing of parallel streams. As a result, the only exchange between the two streams in the channels takes place by diffusion at the interface (Fig. 7a), enabling small analytes to diffuse to the buffer stream, while large particles remain in the sample. A single-layer H-filter (Fig. 7b) was laser engraved and assembled onto a glass substrate as described. A sample containing dye and 20 µm beads to simulate plasma and cellular blood contents respectively was injected into one of the inlets, with DI water used as buffer in the opposite inlet. Device operation was monitored with a microscope camera (Fig. 7c and Supplementary Video S1) at 1 ml hr^−1^ to track the flow splitting between analyte and waste outlets. Samples collected at 5, 10 and 15 ml hr^−1^ from the analyte outlet and analysed by colorimetric assay presented a 19.5% ± 4.3% (n = 4), 13.7% ± 2% (n = 3), and 9.5% ± 2.7% (n = 3) of the dye content of the sample respectively, while a 5.2% ± 1.3% (n = 4), 2.4% ± 0.4% (n = 3) and 5.7% ± 2% (n = 3, all data presented as mean ± s.e.m) of the particles was collected respectively in the analyte outlet as contamination (Fig. 7d). A two-way mixed ANOVA analysis revealed a significant difference between bead contamination and dye content for the 5 ml hr^−1^ flow rate (**p < 0.01) and the 10 ml hr^−1^ flow rate (*p < 0.05), while no difference between analyte and contamination was found in the 15 ml hr^−1^ case.

A T-junction droplet generator is a two-phase flow microfluidic device that exploits hydrodynamic forces that arise at the intersection of two channels containing immiscible fluids to generate droplets of a dispersed phase in a continuous phase (Fig. 7e). A droplet generator was designed and fabricated as described (Fig. 7f), with a central channel width (Wc) of 1.32 ± 0.06 mm and a side channel of width (Wd) 333 ± 52 µm intersecting the main channel at a 90° angle, resulting in a width ratio (Wd/Wc) of 0.25 ± 0.04 (mean ± s.e.m, n = 3). Stable generation of water-in-oil droplets (Fig. 7g) was achieved with a constant flow rate ratio (${Q}_{C}/{Q}_{D}$) of 1.63 and dispersed-phase flow rates of 0.2 ml hr^−1^ (Supplementary Video S2), 0.5 ml hr^−1^ and 1 ml hr^−1^, resulting in droplets with diameters of 741 ± 24 µm (mean ± SD, n = 33), 606 ± 20 µm (mean ± SD, n = 26), and 533 ± 17 µm (mean ± SD, n = 70) respectively, and polydispersity index of 3.24%, 3.35% and 3.11% respectively (Fig. 7h).

## Discussion

The requirement for complex photolithography equipment and clean room facilities poses an entry barrier for the use of microfluidic tools in biomedical research. Moreover, the high cost of photomasks often hinders rapid iteration of microfluidic designs. Thermoplastic materials offer a number of advantages with respect to PDMS soft-lithography for rapid, low cost microfluidics due to their better mechanical properties, hydrophilicity and versatility in design and fabrication. The use of PMMA and other thermoplastics enables both iterative rapid prototyping and industrial manufacturing, accelerating the design of a prototype and the translation to a final product with a clinical or commercial application. Here, we have reported a laser-micromachining fabrication and adhesive-based assembly method for PMMA microfluidics that requires little expertise in microfabrication techniques and can be achieved with low cost and readily available equipment and reagents.

The use of laser micromachining offers a high versatility, both in terms of device design and channel geometry, without the need for a photomask or microfluidic mould. Here, we have shown that control of laser parameters can result in a wide range of dimensions and aspect ratios with applicability in microfluidics spanning several orders of magnitude. The two parameters studied here, laser power and distance-to-focus, represent the amount of energy and its distribution or spread respectively. Higher power increased the penetration of the beam into the material, resulting in the observed increase in depth, while DF increases the spread of energy over a wider region and in turn decreases the energy density of the laser, resulting in shallower and wider channels as found by both profilometry and scanning electron microscopy. The increase in width with increasing power suggests that, at low powers, a fraction of the laser spot has insufficient energy to cause ablation of the PMMA. Moreover, this effect was found to plateau at high power, consistent with the fact that, at these higher powers, the entire laser spot has enough energy to cause ablation.

The high surface roughness resulting from the laser ablation may be one of the main problems preventing the widespread application of laser-machined PMMA devices in microfluidics. Here, we have shown that a significant improvement on surface quality can be accomplished with a combination of vapour solvent surface treatment and low temperature annealing. While careful control of the treatment times and exposure temperature is critical to achieve a balance between surface improvement and crack formation, an optimal surface quality can be obtained without altering device architecture.

In contrast to previous studies that utilise solvent vapour surface treatment, which typically report room temperature treatments^13,15^, our results suggest that accurate temperature control during the solvent vapour exposure process is critical to obtain a reproducible remodelling of the surface. We demonstrate that for the same treatment time, a temperature difference of 5 °C results in observable differences on the surface quality, likely due to the temperature dependency of the solvent evaporation rate.

Except one of the treatment configurations (10 minutes exposure at 25 °C), the combination of acetone vapour and thermal treatment did not result in a significant deformation of the device architecture, with changes in dimensions below 5% after reflowing of the surface thermoplastic material. The formation of cracks and wrinkles observed on the surface of the treated channels was more evident at longer treatment times and higher exposure temperatures. This crack formation is consistent with the combination of thermal and solvent-induced stresses caused during the treatment, a phenomenon that is well characterised in PMMA and other glassy polymers. However, under some treatment configurations (e.g. 3 minutes exposure at 30 °C), a smooth channel surface was achieved without observable cracks.

The contact angle of PMMA was found to increase upon laser ablation as previously reported^25^. The increase in contact angle after surface treatment could be an indication of decreased surface roughness as some previous studies report a decrease with increasing surface roughness^26^. However, it has been proposed that laser-ablated PMMA follows an intermediate Wenzel/Cassie-Baxter model^25^ and the interaction between contact angle and surface roughness may be complex. Moreover, the differences observed between non-treated and solvent-treated contact angle could be caused by changes in surface chemistry. De Marco *et al*. reported that a similar surface treatment decreases the contact angle^13^ of femtosecond-laser ablated PMMA, contrary to our findings. The differences in energy between femtosecond pulsed lasers and CO_2_ lasers result in different forms of ablation, namely photochemical and photothermal degradation respectively. The resulting differences in surface texture and chemistry caused by femtosecond-pulsed lasers *versus* CO_2_ lasers could explain this difference in observations^27^. In all cases, the contact angle measured is lower (i.e. higher hydrophilicity) than typical values reported for PDMS (>105°)^28^.

Although the surface quality achieved with this treatment is still lower than glass and PDMS devices, which will continue to be the material of choice for optofluidic applications, the surface quality improvement enables visualization inside the channels, which is normally reduced due to surface imperfections (Supplementary Fig. S2a). The surface treatment method presented here can be carried out without the need for complex equipment and acetone was selected for its high solvent power, relative safety and availability. This technique was found to be more effective in achieving surface remodelling without warping than conventional thermal fusion (Supplementary Fig. S2b) for the same treatment time. The simplicity and accessibility of this technique, and its compatibility with high-throughput industrial manufacturing, are key to facilitate the transition of these technologies from concept to prototype and their translation to clinical and consumer products.

The adhesive bonding method developed here to assemble the devices produces a tight seal between the substrate and the chip, even if the surfaces cannot achieve conformal contact due to irregularities, and the capillarity-driven method resulted in uniform adhesive application as opposed to stamping and rolling. In this method, the capillarity forces that arise at the microscopic interstitial space between the chip and the substrate drive the flow and expansion of the adhesive mix, while the sudden drop in the driving force due to the change in height at the edges of the channels prevents channel clogging by arresting the flow of adhesive. The adhesive bond was stable at the range of temperatures typically required in microfluidic applications (room temperature to 37 °C), but became unstable at high temperatures (~80 °C), causing delamination between the chip and the substrate. While this enables recycling of the PMMA device and repositioning of the substrate, it can limit high temperature applications such as continuous-flow PCR. Other commercially-available epoxy adhesives may be able to withstand higher temperatures and could be explored for these applications.

This method also enables more versatility than the conventional thermal fusion. We demonstrated the compatibility of this bonding method with conventional substrates (PMMA and glass) as well as silicon and a piezoelectric material (LiNbO_3_). This higher material flexibility enables a wider range of applications for these PMMA microfluidic devices that are usually restricted by the material limitations of conventional thermal fusion bonding, including electrochemical and acoustofluidic applications.

The accumulation of adhesive and its shape at the interface has been observed in similar bonding setups^29^. This conformation could be the result of surface tension at the borders of the channel, where the drop in capillary pressure halts the flow of the adhesive generating a concave meniscus. While this accumulation does not interfere with conventional applications, it could be exploited to generate channels of circular cross-section, something difficult to achieve with PDMS^30–32^. The bonding strength resulting from this adhesive delivery method was analysed using a standard test (ASTM D3163) for lap-shear joints. For samples cured for 72 hours, the presence of residual adhesiveness in the overlapping area after debonding suggest incomplete curing of the adhesive within this time frame, resulting in the lower bond strengths observed here. On the other hand, samples stored for 2 months remain bonded and display a higher bonding strength, making this bonding technique suitable for long term storage of microfluidic devices in commercial or clinical applications. The bond strength resulting from this method was found to be lower than typical bond strengths reported for thermal fusion and solvent-assisted thermal fusion (~2–20 MPa)^33,34^, likely due to the indirect nature of the bond, but higher than reported adhesive bonding strengths^23^ and some thermal fusion bonding strengths (130 kPa)^35^.

Capillarity-assisted adhesive bonding presents two main limitations, namely adhesive accessibility and bubble generation. First, microfluidic “islands”, i.e. surfaces that are completely enclosed by channels, cannot be accessed by the adhesive through capillarity-driven flow. This limitation can be overcome by adding laser-cut access holes to the design of the islands, enabling direct delivery of the adhesive (Supplementary Fig. S5), although this solution is constrained by the available space for the access holes, as small holes were found to cause failure of the bonding process (Supplementary Fig. S3b). The second complication is the formation of small bubbles in the interstitial space in devices with larger bonding areas due to irregularities in one or both of the surfaces that prevent adequate capillary filling. These bubbles were found to have no effect on the bonding process and no leaking was observed even when bubbles are present. A uniform adhesive layer devoid of bubbles was achieved by designing channels with thin walls (~500 μm), which can be critical for some applications, including acoustophoresis. The simplicity, versatility and accessibility of this method make it a strong candidate for assembly and packaging of thermoplastic microfluidics, and future development should aim at increasing the throughput of this bonding technique to make it more compatible with industrial manufacturing.

The applicability of this fabrication method was demonstrated with two common microfluidic devices. The H filter was applied as a size-fractioning system to separate a sample containing blue dye (molecular weight 825.97 g mol^−1^) and micron-sized beads (diameter 20 µm) to emulate plasma and cellular contents of blood respectively. The differences in diffusion coefficient between the dye (estimated in the order of 10^−6^ cm^2^ s^−1^ based on similar dyes^36^) and the particles (estimated in the order of 10^−10^ cm^2^ s^−1^ based on their size) enable diffusion of the smaller species (dye) to the buffer stream and collection in the analyte stream, while the low-diffusivity beads are eliminated in the waste outlet. At 5 ml hr^−1^ and 10 ml hr^−1^, the blue dye was enriched in the analyte stream, while no significant difference was found at higher flow rates. No significant difference in the bead contamination was found for the different flow rates, suggesting it could be caused by intrinsic characteristics of the device and not by diffusion into the neighbouring stream. In this context lower flow rates favour dye purification, as the transient time in the device increases, enabling higher mass transfer of the dye by diffusion without additional contamination. However, sample collection at lower flow rates was hampered due to differences in hydraulic resistance of the microfluidic tubing connected to each of the outlets, resulting in flow instabilities at the splitting point of the two streams.

The T-junction achieved water-in-oil droplet formation in a dripping regime at all flow rates explored (0.2, 0.5 and 1 ml hr^−1^), due to the flow rate ratio and width ratio between the central and side channels, while the large dimensions of the central channel (>1 mm) prevent droplet formation via squeezing^37^. The droplets generated presented a low polydispersity index (~3%), comparable to other T-junction devices^38^ and other microfluidic emulsion generators^39^. The droplet diameter was also found to decrease with increasing flow rate, consistent with other reports and theoretical modelling of droplet generation at high capillary numbers (ratio between shear forces and interfacial tension) that characterise the dripping regime^40,41^.

The two microfluidics devices presented here illustrate how this technique can be applied to fabricate devices in a cost-effective and accessible manner: the devices were fabricated for a low cost (<1£ per device) and engraved at a fast rate (<30 seconds per device), and using readily available material and equipment. Both the H-filter and the T-junction are simple and commonplace devices that can be used as building block of more elaborate microfluidic systems with complex architectures. For the H-filter, chip-to-world interfacing was accomplished by pressure-fitting conventional flexible microfluidic tubing to laser-cut access holes in the devices. In the case of the T-junction device, laser-cut PMMA rings were used to interface the devices’ ports with the tubing. This construction enabled gluing of the tubing to the rings and the rings to the main device without clogging the tubing, producing a more durable seal that can withstand the higher pressures produced when pumping oil through the device. The flexibility of the laser-machining method allows for custom-sized holes to be easily incorporated into the chip design to accommodate for tubing with a variety of external diameters. Moreover, the robust mechanical properties of PMMA could enable standard microfluidic connections and adaptors, including luer lock and upchurch nanoport connectors, to be readily integrated into the chip for simple, plug-and-play microfluidics.

## Conclusion

Thermoplastics are emerging as a substitute for conventional materials in microfluidics devices with a commercial or clinical application owing to their higher versatility, low cost, industrial scalability and ease of handling and packaging. Here we have presented a method for fabrication and assembly of inexpensive PMMA microfluidic devices. We have demonstrated that laser ablation is a suitable technique for mask-less rapid prototyping with high design flexibility, enabling the engraving of channels with a variety of dimensions, aspect ratios and morphologies by controlling laser parameters. A combination of acetone vapour and thermal annealing was found to effectively reduce the surface roughness caused by the ablation process, although control of exposure time and temperature is critical to ensure reproducibility and minimise crack formation. Moreover, the capillarity-assisted adhesive bonding developed here allows for room temperature bonding of PMMA with a variety of materials, even when surface irregularities are present.

The accessibility and flexibility of this method, as shown by our example microfluidic devices, combined with the ease of sharing computer designs, could open a new avenue towards cost-effective microfluidic technologies with a high translation potential from the benchtop to the clinic.

## Electronic supplementary material

Supplementary Material

Supplementary Video 1

Supplementary Video 2
