# Supplementary material for: Cost-effective rapid prototyping and assembly of poly(methyl methacrylate) microfluidic devices
Source: Sci Rep. 2018 May 3;8:6971. doi: 10.1038/s41598-018-25202-4 (PMC5934357; doi:10.1038/s41598-018-25202-4)
Supplement: Supplementary file 1 — Supplementary Material [file 41598_2018_25202_MOESM1_ESM.docx]

**Supplementary Material**


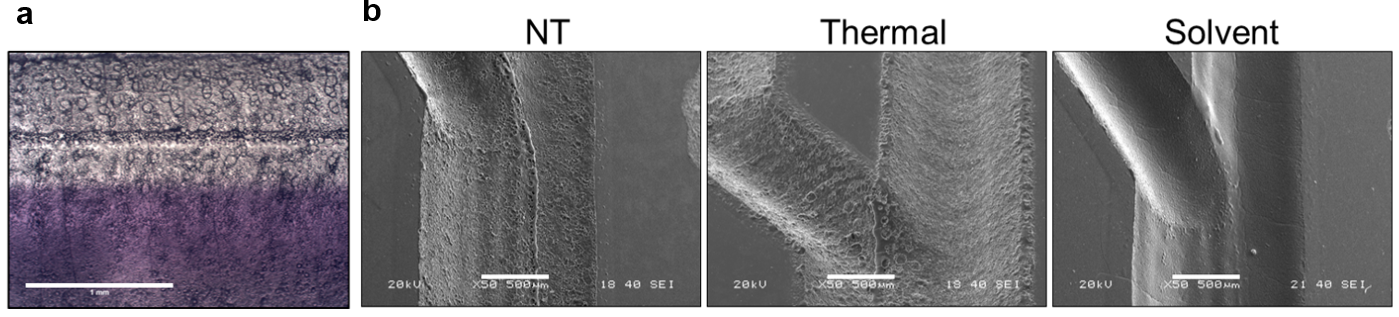


**Supplementary Figure S2. Surface treatment methods and visualisation**. (a) Non-treated microfluidic device with two parallel streams (blue dye and deionised water). The presence of pores, a central ridge and other imperfections limits the visilibility in the channel. Scale bar: 1 mm. (b) Non-treated samples (NT), samples heat-treated at 110°C for 30 minutes (thermal) and samples treated with acetone vapour at 25°C and heat threated for 20 minutes at 70°C (Solvent) were analysed by scanning electron microscopy. Scale bar: 500 μm.

**Supplementary Figure S1.** Analysis of channel width changes after acetone exposure at 30°C for different treatment times followed by thermal remodelling. No significant differences exists between the untreated sample and any of the treated samples. Data analysed by one-way ANOVA with Tukey post-hoc test. (Mean ± s.e.m, n=3)

**Supplementary Figure S3. Stamping transfer adhesive bonding.** (a) Schematic of the adhesive delivery method. A thin layer of adhesive is spread on a sacrificial surface, and the engraved device is stamped on this layer and transferred to the final substrate. (b) This technique is prone to channel clogging (white arrow) due to excess adhesive. Scale bar: 1 cm (c) Non-uniform coating of the adhesive layer results in channel leaking (black arrow). Scale bar: 1 cm. (d) Device with multiple holes for advesive injection (white arrows). While this method enables automated injection of a standard adhesive volume, the presence of multiple holes of small diameter limits the flow of the adhesive due to their intrinsic capillarity and introduces bubbles in the interface, making these devices prone to leaking and spilling.

**Supplementary Figure S4.** Failure load during lap-shear joint tensting of PMMA samples bonded via adhesive bonding (cured for 72 hr or aged 2 months) and via solvent-assisted thermal bonding. Adhesive bonded specimens presented failure at the bonding surface, whereas thermally bonded specimens dailed due to fracture of the material. ** p= 0.01, *** p=0.001. (Mean ± SD, n=3)

**Supplementary Figure S5. Example microfluidic device with a microfluidic island.** A laser-cut access hole enables direct delivery of the adhesive to the island during bonding (a) Computer design of the device, the access hole is represented in green (b) PMMA device with an access hole (white arrow) to enable microfluidic island bonding. Expanding adhesive fronts (yellow arrow) can be observed in the island. Scale bar: 1 cm
